# Fungi and Disease

**Published:** 1868-10

**Authors:** 


Fungi and Disease.—The question which is now on the *tapis* in professional circles, and which it is of the highest interest to obtain a satisfactory reply to, is—In how far do fungi and disease stand to each other in the relation of cause to effect? Professor Hallier, Mr. Simon, Dr. Salisbury, and all that school, believe implicitly in the influence of fungi as causes of disease. On the other side, we have two very able authorities in this country in Dr. Thudichum and the Rev. J. M. Berkeley, who utterly deny the fungus hypothesis. Now, it must be admitted that up to this time the advocates of the fungus theory, on whom the *onus probandi* fairly lies, have failed in all cases to do more than show the coincidence of fungi and disease. But this is but small ground for the inference they draw from the observation. As has been often suggested, both the disease and the fungus may be coincident terms of the same unknown condition. The crucial test is a tolerably easy one. Let Dr. Salisbury and his party propagate their fungi, and then, by inoculation, reproduce the disease with which the parent fungi were originally associated. This would convince every one. But really, till it is accomplished, it is unwise to push too far a fascinating hypothesis, which too sanguine practitioners may make the basis of an unsound and therefore dangerous practice.—*Medical Times and Gazette.—News and Library.*

